# Improving the quality of antenatal screening and early intervention for alcohol and other drug use: protocol for a multi-stage approach to systems reform

**DOI:** 10.1186/s13722-023-00434-6

**Published:** 2024-01-05

**Authors:** Matthew W. R. Stevens, Megan Cooper, Lynette Cusack, Robert L. Ali, Annette L. Briley

**Affiliations:** 1 School of Biomedicine, The University of Adelaide, Adelaide, South Australia Australia; 2 Caring Futures Institute Flinders University, Bedford Park, South Australia Australia; 3 Adelaide Nursing School, The University of Adelaide, Adelaide, South Australia Australia; 4 Northern Adelaide Local Health Network, Lyell McEwin Hospital, Elizabeth, South Australia Australia

## Abstract

**Background:**

Alcohol, tobacco and illicit drug use during pregnancy can cause significant harm to women and their developing fetuses. Despite recommendations for abstinence during pregnancy, some women continue to use, making screening for substance use during antenatal clinic attendances an important strategy for reducing risk. This study aims to improve the rates of screening and intervention for substance use among pregnant women, including appropriate referral for those who may be substance-dependent. The protocol outlined here focuses on a multi-stage implementation study.

**Methods:**

This study will occur in four phases. Phase 1 will identify a baseline rate of screening and subsequent care at the antenatal clinics of two, South Australian hospital-based maternity services, through a retrospective case note audit. Rates of self-reported substance use identified in the case notes will also be compared against representative data from Adelaide Primary Health Network to establish rates of over or underreporting. Phase 2 will involve an online Training Needs Analysis of midwifery staff working at those services, to assess their knowledge, attitudes, beliefs, and commitment to the care of women who use substances during pregnancy. Phase 3 will involve a training package for all midwifery staff at those services, focused on routine screening for substance use, and how to provide appropriate care. Outcome measures from phase 2 will be reassessed during phase 3 and any changes since training will be evaluated. Phase 4 will then repeat phase 1 to compare the changes in rates of both screening and any associated intervention before and after training.

**Discussion:**

From a public health perspective, this project has the potential to make a significant impact on reducing risk of harm from substance use disorders among pregnant women, and contribute to better health outcomes for their children.

*Trial registration: *This trial has been pre-registered under the Open Science Framework. Registration: 10.17605/OSF.IO/73FDZ.

## A note on terminology

The authors recognize that *gender* is a diverse, social construct encompassing a person’s identity, and therefore not all individuals who give birth will identify as *women*, or *mothers*. *Sex*, on the other hand, often refers to biological attributes which include physical anatomy (internal and external), hormones, and chromosomal type. Given this protocol focuses on issues in which the individual’s *sex* is central, we will refer to *women who are pregnant*, or *women who give birth*, in reference to the individual’s biological sex. This decision was also taken to avoid unintended harmful consequences from using language that can be dehumanising, disembodying, and ultimately disempowering to women.

## Introduction

Alcohol, tobacco or other substance use during pregnancy is harmful to fetal and child development. Alcohol consumption increases the risk of miscarriage, stillbirth, low birth weight, and Fetal Alcohol Spectrum Disorders (FASD) [1–5]. FASDs are the leading preventable cause of non-genetic developmental disability in Australia [6]. To mitigate these risks, the Australian National Health and Medical Research Council (NHMRC) guidelines recommend that women abstain from alcohol when planning a pregnancy, during pregnancy, or while breastfeeding [7]. Despite this guidance, previous studies indicate low adherence to this advice, with many women continuing to drink alcohol after confirming their pregnancy [8, 9].

Tobacco smoking during pregnancy is also linked to a range of negative outcomes, including low birth weight, small for gestational age, preterm birth, and sudden infant death syndrome (SIDS) [10–12]. Smoking is the most common preventable risk factor for pregnancy complications [13]. However, in 2019, 9.2% of Australian women reported smoking during pregnancy [14]. Illicit drug use during pregnancy (including cannabis, amphetamines, and opioids) also places both the woman and developing child at increased risk of harm and adverse outcomes [15–18].

Antenatal clinics offer an ideal opportunity to identify and address substance use during pregnancy. The World Health Organization (WHO) recommends screening all pregnant women, and offering brief interventions for those who continue substance use [19]. Evidence suggests that this approach increases the likelihood of abstinence among pregnant women [20, 21]. Despite being part of midwives’ public health role for some time, there are a range of barriers which can impact the effectiveness of implementation. Structural-level factors (e.g., time, knowledge, resource constraints) affect the likelihood that screening will occur in the first place [22, 23], while a range of psychosocial factors (e.g., stigma, shame, guilt, legal consequences) create barriers to disclosure [24]. Knowledge, skills and attitude gaps can be addressed through training, but evidence suggests that many such interventions will fail during implementation, unless other structural barriers are adequately addressed [25]. Therefore, overcoming these challenges requires a multifaceted approach, which not only seeks to build the capacity of midwives to undertake the work effectively, but also targets these structures and systems required to support them.

Introducing a valid and reliable screening tool for substance use into the antenatal assessment battery is necessary for systems reform. While a number of instruments for single substances are available, options for screening multiple drugs are limited. ASSIST-Lite is a tablet-based screening tool which quantifies risk of harm for a range of common substances, and can guide brief advice or facilitate active referral depending on the level of risk [26]. Although ASSIST-Lite was designed, and has been validated, for use in time-critical areas (e.g., emergency departments) [26, 27] to our knowledge it has not been validated for use amongst pregnant women. Despite this lack of evaluation in the antenatal setting, ASSIST-Lite is currently included in the standardised antenatal assessment at one of the largest metropolitan tertiary referral centres in South Australia. Given that there is no safe level of substance use during pregnancy, modifications were made to the cut-off scores to reduce risk thresholds, so that any level of use would be a target for appropriate intervention or referral. However, before the instrument can be fully implemented across the state’s other metropolitan antenatal services, it is necessary to examine current screening practices in those sites, as well as potential facilitators and barriers that might impact implementation.

The overall aim of this study is to contribute to the reduction of harm associated with substance use during pregnancy, and the early postpartum period. This involves increasing the rate of screening for alcohol and other drugs (where screening is not universally applied); improving the quality of advice, care and support for women using substances to achieve abstinence; and finally by improving integrated care pathways with specialist treatment services for women who are at high-risk of dependence. It is anticipated that supporting women to achieve abstinence during pregnancy will lead to healthier fetal and child development and ameliorate the short and long term associated health and societal impacts of these lifestyle choices. The research study comprises four phases: a retrospective case note audit, a training needs analysis of midwives’ knowledge, attitudes, beliefs and commitment to caring for pregnant women who use drugs, both before, and after a training intervention, and a six-month follow-up case note audit. Qualitative data, including online surveys will also be collected to gain deeper insights into facilitators and barriers influencing midwives’ screening practices.

## Methods/design

### Study design

#### Phase 1: retrospective case note audit

In the initial phase of this study, we will randomly select one hundred sets of case notes from maternity records at tertiary maternity services in South Australia. Records will pertain to women attending for their first antenatal visit between April and September, 2019. Rates of self-reported use will be compared to nationally representative data from the National Drug Strategy Household Survey 2019 as a benchmark [14]. The period chosen (i.e., April to September, 2019) allows us to evaluate the levels under- or overreporting identified in our sample.

We will exclude cases from women who transferred care to either participating site later in pregnancy, or did not receive antenatal care at either site, despite giving birth within the maternity service. For clarity, all inpatient antenatal, intrapartum and postnatal care are provided at the Lyell McEwin Hospital, though some women are seen for antenatal care at Modbury Hospital. Case notes will be randomly selected using Unit Record (UR) numbers from the computer system.

Minimal demographic information will be extracted from the selected case notes, focusing on screening data related to alcohol, tobacco, and other drug use before and during the current pregnancy, along with details of any intervention. Extracted data will be limited to maternal age (given as month, and year of birth), maternal ethnicity, parity, date of the first antenatal appointment, estimated date of delivery (EDD), documented screening for previous and current alcohol, tobacco (including e-cigarettes), amphetamine-type stimulants, cannabis and prescription drug use. Details of who documented the history (e.g., midwife, nurse), and any action taken in response to disclosure will also be extracted.

Two of the principal investigators AB and MC (both midwives registered with Ahpra), will oversee privacy and confidentiality during data collection. Each investigator will review the complete set of case notes, and a third reviewer (LC) will cross-check the data separately for consistency and reliability. Any disagreements will be settled by the third reviewer.

Five batches of 20 case notes will be requested from Medical Records personnel at the Lyell McEwin (where all medical records are stored), with a minimum of 2 days’ notice. All notes will be reviewed in a private office, adhering to local policy and practice for secure handling. Locked filing cabinets will be used to store the notes, and access will be restricted to the research team. All collected data will be anonymized, assigning each case a unique ID. Only the month and year of maternal date of birth will be recorded to further ensure anonymity. Confidentiality will be maintained at all times.

Data will be extracted into an audit-specific proforma, and the information will be entered into a password-protected Excel spreadsheet. Reported rates of previous (i.e., lifetime) and current substance use will be compared to rates from the 2019 National Drug Strategy Household Survey (NDSHS), focusing on pregnant women in Adelaide, as reported by the Australian Institute of Health and Welfare (AIHW) [14].

#### Phase 2: training needs analysis

After reviewing the case notes, we will invite a subset of midwives who are responsible for antenatal booking assessments to participate in an online, Training Needs Analysis (TNA) survey. The survey will be designed to assess knowledge, attitudes, beliefs, and commitment to care regarding the assessment and management of women who use substances during pregnancy. This will also include questions around nicotine vaping. Findings from the survey will be used to inform the development of the training program, to be delivered during phase 3. Northern Adelaide Local Health Network (NALHN) have requested that the survey remain anonymous due to concerns about potential identifiability among the small sample of eligible midwives.

To be eligible for phase 2, midwives must currently work on rotation in clinical areas within NALHN, and specifically involved in provision of antenatal care. This represents a potential sample of approximately 30 midwives (at any given time and including all models of care delivery). Midwifery students and midwives not working in the antenatal areas will be not be eligible. Before enrolment, eligible midwives will receive an information leaflet outlining the survey’s aims and objectives, and the nature of voluntary participation. Confidentiality of their involvement will also be discussed, and ample opportunity to ask questions, and have them answered to their satisfaction will be provided. Midwives will be informed of their right to decline participation, or the right to withdraw at any time without penalty. Informed consent is a requirement of participation.

#### Phase 3: training implementation and second survey

Phase three will begin once results from the TNA have been reviewed. In this phase, all midwives, including student midwives, those not working in clinical areas, and those responsible for antenatal booking assessments at will undergo an in-service training package aimed at enhancing their knowledge, skills and confidence in identifying and responding to substance use during pregnancy. Participation in training will be a requirement by NALHN Women’s and Children’s Division, and will occur during regular professional development hours. However, only those who are responsible for booking assessments (approximately 30 at any given time) will be invited to complete a re-evaluation survey from phase 2. Once more, the survey will be anonymous to protect midwives from potential identifiability from small numbers, and informed consent will be required before participation undertake the second survey.

##### *Training intervention*

The training package will cover four broad objectives. First, it will raise general knowledge and awareness of the risks and harms associated with substance use during pregnancy, both for the mother and developing fetus. Second, it will teach how to effectively use the ASSIST-Lite, which will be included into the clinical information system across both services, to identify women at risk of harm or dependence. Third, it will provide guidance on offering effective brief advice around abstinence to pregnant women who are using, but who are not at high risk. Finally, it will clarify integrated care pathways with Drug and Alcohol Services South Australia (DASSA) for women at high-risk of dependence.

Two registered midwives (AB, MC), and a public health physician/addiction medicine specialist (RA) will design and deliver the training. A clinical nurse from DASSA will also be present to discuss the process of actively referring higher risk cases. The training will be accredited for professional development hours, and will consist of two sessions, each lasting approximately 60 minutes each. These will follow the format previously used in an implementation study of ASSIST-Lite in the Emergency Department of a large metropolitan health service [27]. The training will be tailored to address any gaps identified through phase 2. It is expected that multiple instances of the training program will be required to cover all midwives working in the service.

##### *Post-training survey*

Following the training, the anonymous online survey will be repeated (from phase 2) to re-assess midwives’ knowledge, attitudes, beliefs and commitment to care for pregnant women who use alcohol or other drugs during pregnancy. All midwives who undertake the training will be invited to complete the second survey. We will include an open-ended item on the survey to identify the proportion of midwives who completed both surveys (phase 2 and phase 3; e.g., “*how many times have you completed this survey*?”). This will enable quantification of any group-level changes that may have occurred in response to the training.

#### Phase 4: second retrospective case note audit

The final phase of this research will involve a follow-up case note audit to determine if the training has influenced the rates of screening for alcohol, tobacco and other drug use. The second retrospective audit will take place at least six-months after the training, and will include a random sample of one-hundred sets of records from the previous six-month period. To account for seasonal effects, we will use a data-collection window consistent with phase 1 (i.e., April to September). The same data extraction tool and methodology used in phase 1 will be employed in phase 4. Data collected during phase 4 will be compared to the frequency and level of detail provided in the case notes sampled during Phase 1. Similar to phase 1, reported rates will be compared with the most recent AIHW data when it becomes available.

### Ethical considerations

For a number of practical reasons, informed consent will not be required for phases 1 and 4 of this project, as they represent a retrospective review of the case notes. All case notes will be de-identified during the data extraction process. As such, this study poses no discernible risks to participants, as all subjects will have completed the episode of care, and care pathways for mothers and infants prior to case note review, and therefore their care remains unaffected by inclusion in this study. Obtaining consent from women no longer engaged with maternity services would prove impractical and all results presented will be anonymised.

The findings from this study are highly unlikely to have any significance for the participants’ welfare, as their maternity episode will have already been completed before case note review is undertaken. The data from this study will not be exploited commercially, and participants will not be deprived of any potential financial benefits.

### Measures

 The TNA online surveys used in phases two and three of this study, will consist of a variety of self-administered, standardized questionnaires that assess knowledge, skills, attitudes, beliefs and commitment to care for women who use alcohol or other drugs during pregnancy. The wording in the questionnaires will be adapted to suit a midwifery audience. For example, some scales which refer to specific drugs (e.g., alcohol) will be reframed to focus on *substance use*, while scales that use outdated language (e.g., abuse, *addict*) will be updated to reflect contemporary terminology (e.g., dependence, woman living with dependence). All scales will be standardized and scoring algorithms re-calibrated to a 7-point Likert scale, ranging from (-3) strongly disagree to (3) strongly agree. As such, a score of zero will imply neutral agreement. The following standardized measures will be used:

### Medical condition regard scale (MCRS)

The Medical Condition Regard Scale (MCRS) is a 12-item scale designed to assess a range of stigmatic attitudes towards people with a variety of medical conditions [28]. For this study, the MCRS will be split into three separate questionnaires which assessed midwives’ attitudes towards women who use alcohol, tobacco and illicit drugs while pregnant respectively. The scale has been used previously and has been validated for use in nursing and midwifery contexts [29, 30].

### Substance abuse attitudes survey (SAAS)

For the purposes of this study, a shortened version of the Substance Abuse Attitudes Survey (SAAS) will be used [31]. The original SAAS is a 25-item survey designed to assess a range of attitudes and beliefs about people with substance use disorders. For this study, a condensed, 16-item version of the instrument will be used.

### Drug problems and perceptions questionnaire (DPPQ)

Attitudes and commitment to care for women who use drugs will be assessed using the 18-item Drug Problems and Perceptions Questionnaire (DPPQ) [32]. DPPQ is adapted from the Alcohol and Alcohol Problems Perceptions Questionnaire (APPQ). The scale has been used previously and has been validated for use among healthcare professionals [31].

### Shortened alcohol problems and perceptions questionnaire (SAAPPQ)

Attitudes and commitment towards working with people who drink alcohol during pregnancy will be assessed using the Shortened Alcohol and Alcohol Problems and Perceptions Questionnaire (SAAPPQ) [33]. The scale has been used previously and has been validated for use among healthcare professionals [30].

### Short understanding of substance abuse scale (SUSS)

Beliefs about the nature of substance use disorders and women who use drugs during pregnancy will be assessed using the Short Understanding of Substance Abuse Scale (SUSS) [34]. SUSS is a 10-item scale that asks participants to rate their agreement to statements of belief about substance use/dependence and people who use drugs, and has been validated in a variety of contexts, including healthcare [30].

### General self-efficacy scale (GSE)

Self-efficacy will be assessed using the General Self-Efficacy scale [35]. The GSE is a 10-item scale assessing self-efficacy across a broad range of domains. GSE is rated on a 4-point Likert scale ranging from not true at all, to exactly true. Higher scores reflect a greater degree of self-efficacy.

### General tobacco and e-cigarette (vaping) knowledge

Knowledge about the harms related to tobacco use during pregnancy will be assessed through the following questions: ‘what is the level of risk to an unborn child from cigarette smoking during pregnancy?’ for 1–2 cigarettes per day, 3–9 cigarettes per day, and for 10 + cigarettes per day; rated as either harmless, slightly increased risk for the child, or a significantly increased risk for the child. ‘What is the level of risk to an unborn child following changes to cigarette consumption during pregnancy?’ for suddenly ceasing, and for changing to vaping; rated as either, beneficial, no change in risk, slightly increased risk for the child, significantly increased risk for the child. Midwives will also be asked about routine enquiry into smoking, vaping, and partners’ smoking.

### Data analysis

#### Phase 1

Rates of screening (i.e., occurred vs. did not occur [or not reported]) will be established for past and current use, for all substances. These will form the baseline *screening rates* for later comparisons. Differences between screening rates will be analysed with respect to the demographic variables collected. Rates of self-reported alcohol, tobacco and other drug use prior to, and during pregnancy will also reported. These will be expressed as frequencies (and percentages) of total sample, and will form our baseline *prevalence* rates in our sample. Prevalence rates for previous and current use will be compared with respect to maternal age, ethnicity and parity. Student t-tests and non-parametric tests will be used to assess significance of comparisons, with *p*-values below 0.05 indicating statistical significance. Finally, to compare prevalence rates in our sample with rates of use reported in AIHW data, we will use a Bayesian updating approach, which will help to quantify the strength of evidence for any under or overreporting.

#### Phase 2

Midwives’ overall knowledge, attitudes, beliefs, and commitment to care for pregnant women who use alcohol and other drugs will be assessed. Relationships between mediators including self-efficacy, will also be assessed using regression modelling. Average aggregated scores on each of the instruments, with measures of variance (reported as means and standard deviations) will be calculated, and standardisation will occur so that scores centre on a mean of zero. This will help indicate positive or negative sentiment. We will also convert those standardized scores to percentages to determine average agreement or disagreement. Finally, Pearson’s *R* and Spearman’s *Rho* correlation coefficients will be used to explore relationships between measures of knowledge, attitudes, beliefs and commitment to care.

#### Phase 3

Inferential statistics will be used to assess changes in scores before and after the training, using the same survey battery. Since the survey will be anonymous, there will be no way to track individual responses over time. Therefore the analysis will focus on cohort-level changes (discussed later in limitations). The statistical method applied will vary depending on the nature of the data collected. Student t-tests or ANOVAs will be used to compare group means (for 2, and for 3 or more groups respectively), and chi-square tests to compare differences between proportions. Furthermore, an exploratory regression analysis will be used to identify any predictors in our sample for screening rates, based on responses to other items.

#### Phase 4

Rates of screening for alcohol, tobacco and other drug use during pregnancy will be re-assessed. Screening rates will be established by the number (and proportions) of cases where an ASSIST-Lite assessment was recorded. Rates of screening, detection (i.e., prevalence) and level of detail provided for any intervention will be compared to phase 1 using inferential statistics for quantitative data. Changes in note-taking detail/quality will be assessed qualitatively using content analysis. Prevalence rates in phase 4 will also be compared to AIHW data from the most recent NDSHS, using a Bayesian updating approach, which will help to quantify the strength of evidence for any under or overreporting.

## Discussion

This protocol outlines a project aimed at improving rates of screening and detection during routine antenatal care at two metropolitan hospital-based maternity services in South Australia. Given the impact of substance use disorders on health and other perinatal outcomes for women and their babies, prevention and early intervention is a necessary first step in providing support and care for women and their children who may be at risk. This study represents the first of its kind to evaluate the impact of a system-wide implementation of best practices in screening and early intervention in antenatal settings on rates of screening and detection.

Current practices around screening, intervention and referral within the services are not optimized to support women who are using substances during pregnancy. As a result there is the potential that some are navigating pregnancy with a co-existing substance use disorder. It is unlikely that these women are receiving the necessary support and care for themselves or their developing babies. To address the lack of structured approach, this project will oversee the integration of a standardized assessment for quantifying substance use related risk (ASSIST-Lite) into the clinical information systems across both sites. This approach reflects adoption of the model used currently at another large metropolitan tertiary referral centre in South Australia. It is hoped that standardizing care across South Australia will lead to better perinatal outcomes through streamlined referral pathways for women at high risk of dependence.

To achieve our aim, we will provide educational training package to the midwives working in these services, so that they feel adequately equipped to provide support for women with substance use issues in their care. From a public health perspective, this project has the potential to make a significant impact on rates of substance use in general, as well as reduce some of the perinatal harms to developing babies as a result of maternal substance use during pregnancy.

There are a number of strengths with the proposed methodology. First, random selection of case notes during phases 1 and 4 increases the representativeness of the sample to the population. Relatedly, the use of AIHW data from the same Primary Health Network (PHN), and the same time-period provides an objective point of reference to compare rates of detection. Second, the use of standardized measures in the online surveys in phases 2 and 3 provide opportunity to reliably explore midwives’ knowledge, attitudes, and beliefs, and tailor the training to accommodate those needs. Third, using pre-post evaluations will allow us to directly evaluate the training program’s effectiveness on improving rates of screening and detection.

## Limitations

Aside from the strengths of this research there are also some potential limitations that require discussion. First, the retrospective case note audit is limited to data that has been recorded and documented, which may not reflect actual substance use by participants. Moreover, it is generally accepted that underreporting is likely to occur, even with standardized instruments, given all of the other outside factors (e.g., stigma, shame, child custody concerns, etc.). Comparison with benchmark data will help to clarify whether this was the case.

Second, despite the large expected total number of midwives working in the antenatal clinics, relatively few will be eligible to participate in the TNA surveys (e.g., approximately 30). Therefore, the outcomes from the TNA may not be generalisable to the entire population of midwives (e.g., nationally), but will at least be representative of the cohort of midwives working in those services. Relatedly, since all midwives will be required to undertake the training program, the study necessarily lacks a control group. This will limit our ability to establish causal relationships.

Third, due to the anonymized nature of the TNA surveys in phase 2 and 3, there will be no way to reliably track changes in individual scores over time. While this limits the ability to draw causal inferences about the training, we will still be able to assess cohort-level changes across time. The inclusion of an item to identify participants who took part in both surveys will help to provide some clarity on that point.

A final consideration will be the need to ensure the midwives receiving the training are adequately supported in implementing screening and brief intervention practices afterwards, and that knowledge transfers to new midwives entering those services. It is therefore likely that the delivery of additional training modules will be necessary at some point in the future. The appropriate timing of which will be explored after completion of the study.

## Data Availability

Not applicable.

## Abbreviations

AIHW: Australian Institute of Health and Welfare
ANOVA: Analysis of Variance
APPQ: Alcohol Problems and Perceptions Questionnaire
ASSIST: Alcohol Smoking and Substance Involvement Screening Test
DASSA: Drug and Alcohol Services South Australia
DPPQ: Drug Problems and Perceptions Questionnaire
EDD: Estimated date of delivery
FASD: Fetal Alcohol Spectrum Disorder(s)
GSE: General Self-Efficacy Scale
MCRS: Medical Condition Regard Scale
NALHN: Northern Adelaide Local Health Network
NDSHS: National Drug Strategy Household Survey
NHMRC: National Health and Medical Research Council (Australia)
PHN: Primary Health Network
SAAPPQ: Shortened Alcohol Problems and Perceptions Questionnaire
SAAS: Substance Abuse Attitudes Survey
SIDS: Sudden infant death syndrome
SUSS: Short Understanding of Substance Abuse Scale
TNA: Training Needs Analysis
UR: Unit Record [Number]
WHO: World Health Organization
